# 10-Phenyl-9-[2-(tri­fluoro­meth­yl)phen­yl]-3,4,6,7,9,10-hexa­hydro­acridine-1,8(2*H*,5*H*)-dione

**DOI:** 10.1107/S2414314626008254

**Published:** 2026-08-14

**Authors:** Md. Mominuddin, Harendra N. Roy, Pran Gopal Karmaker, Md. Meraz Hossain, Ennio Zangrando

**Affiliations:** a Department of Chemistry, Rajshahi University, Rajshahi-6205, Bangladesh; b Chemical Synthesis and Pollution Control Key Laboratory of Sichuan Provinces College of Chemistry and Chemical Engineering China West Normal University,Nanchong 637002 People’s Republic of China; c Department of Chemical and Pharmaceutical Science, University of Trieste, Italy; University of Aberdeen, United Kingdom

**Keywords:** crystal structure, acridinedione, tri­fluoro­methyl­benzene

## Abstract

The title compound, C_26_H_22_F_3_NO_2_, crystallizes with two independent mol­ecules in the asymmetric unit with very similar geometries. The two mol­ecules are characterized by a short (H⋯F ≃ 2.13 Å) intra­molecular C—H⋯F hydrogen bond between the central methine C atom and the CF_3_ group. The cyclo­hexenone rings adopt envelope conformations, with a maximum deviation of a methyl­ene C atom of 0.55 (6) and 0.63 (5) Å in one mol­ecule and 0.61 (5) and 0.64 (5) Å in the other. The tri­fluoro­methyl­phenyl and phenyl rings are almost normal to the di­hydro­pyridine mean plane, with dihedral angles lying between 84.35 (9) and 88.81 (8)°.

## Abstract 2

The two crystallographic independent mol­ecules in the title compound display short intra­molecular C—H⋯F hydrogen bonds.

## Graphical abstract

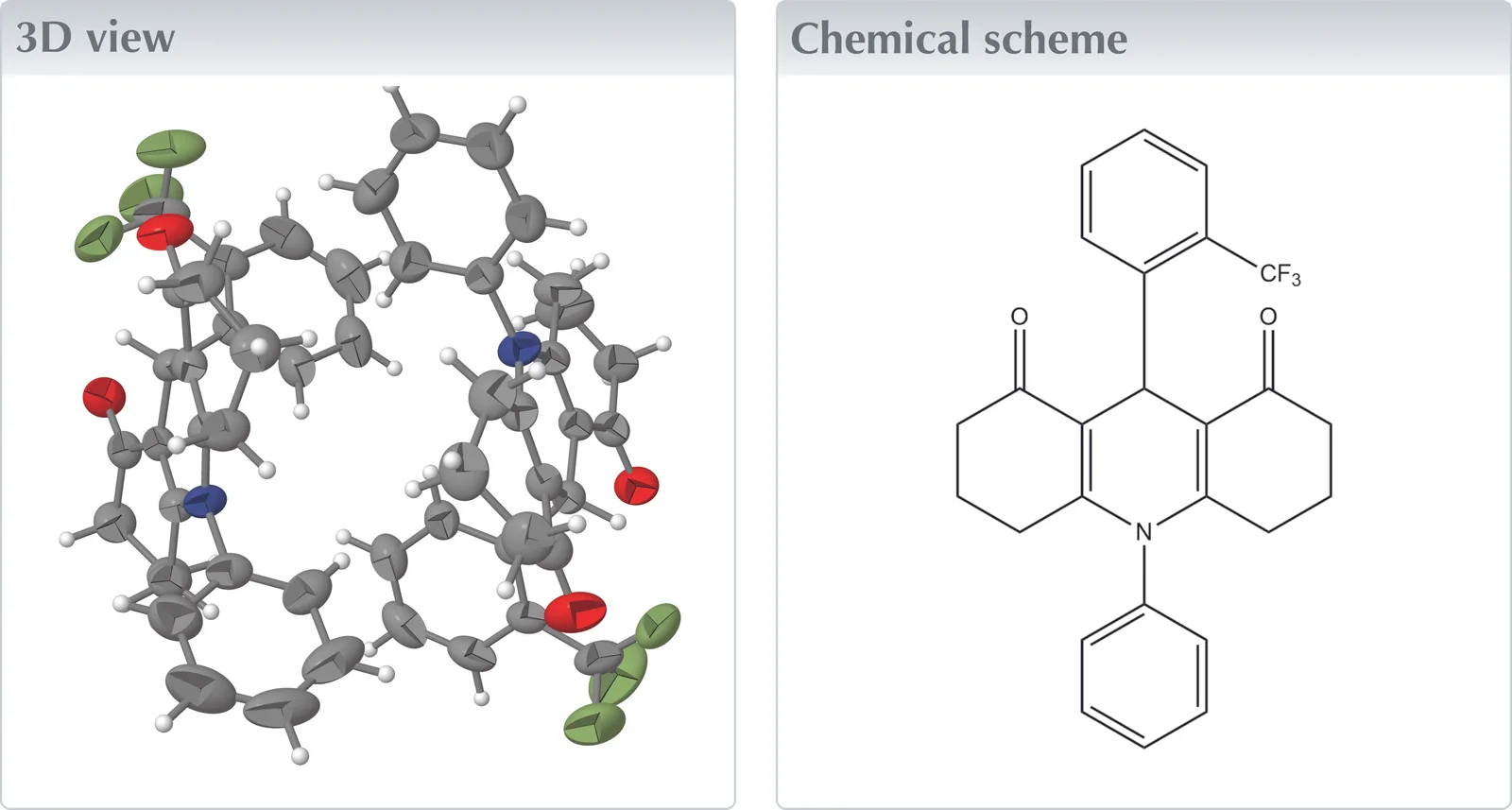

## Structure description

1,8-Acridinedione derivatives are versatile nitro­gen-containing heterocyclic compounds featuring a 1,4-di­hydro­pyridine core. These compounds, which may be prepared by simple syntheses (Venkatesan *et al.* 2008 ▸, Liu *et al.* 2018 ▸) have promise as anti­bacterial (Sirisha *et al.* 2011 ▸) and anti­tumour agents (Ferlin *et al.* 2000 ▸**;** Khatab *et al.* 2023 ▸). In addition, acridinediones have been studied for their photophysical properties as laser dyes (Srividya *et al.* 1998 ▸), as blue light-emitting devices (Islam *et al.* 2003 ▸) and for fluorescence detection of 2,4,6-tri­nitro­phenol **(**Li *et al.* 2024 ▸). As part of our work in this area, we now describe the synthesis and structure of the title compound, C_26_H_22_NO_2_F_3_ (**I**).

**Figure 1:**
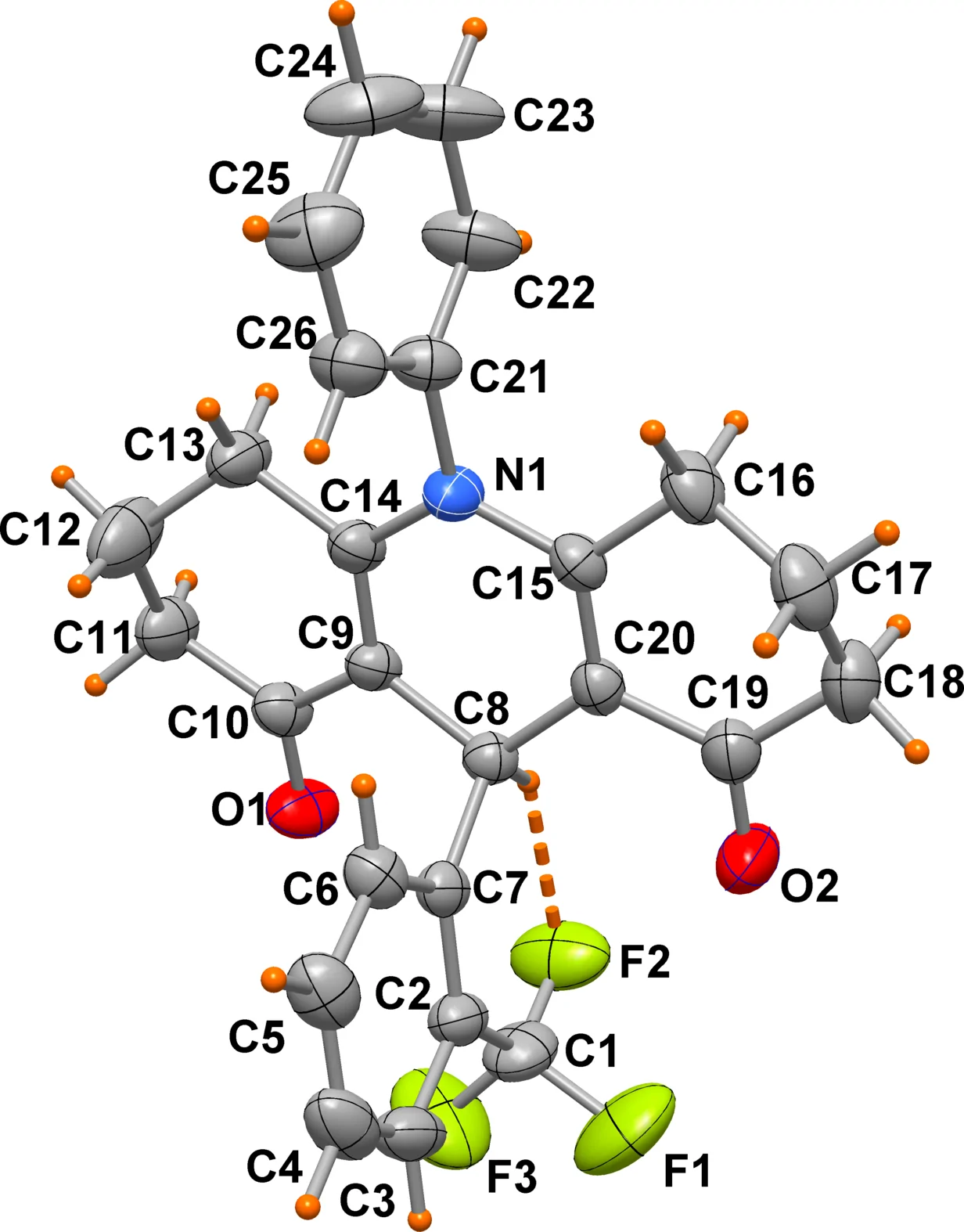
The mol­ecular structure of the first mol­ecule with displacement ellipsoids drawn at the 40% probability level.

The X-ray diffraction analysis revealed that in both independent mol­ecules (Figs. 1 ▸ and 2 ▸) the di­hydro­pyridine ring adopts a flattened boat conformation, the maximum deviation from the di­hydro­pyridine ring being 0.138 (4) Å (C8 in mol­ecule 1) and 0.144 (4) Å (C38 in mol­ecule 2). The two side cyclo­hexenone rings adopt envelope conformations with C12 and C17 (C42 and C47 in the second mol­ecule) as the flap atoms.

**Figure 3:**
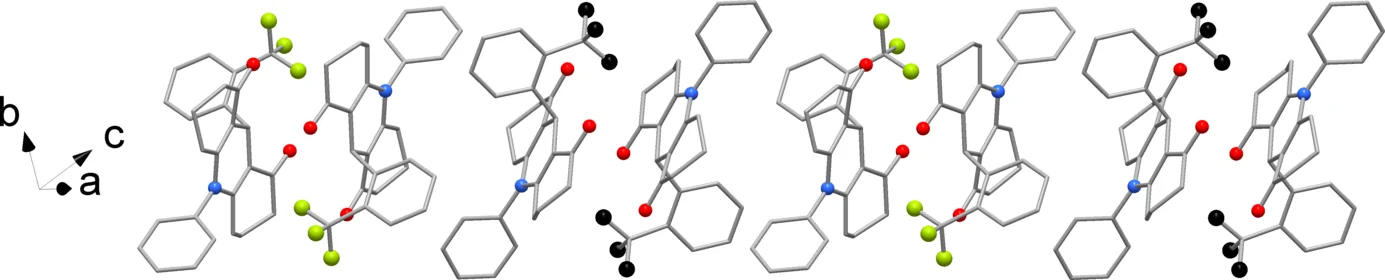
Detail of the crystal packing showing the mol­ecules arranged in pairs in the [1
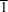
1] direction (black spheres are F atoms of mol­ecule 1).

The tri­fluoro­methyl­phenyl (C2–C7 and C32–C37) and phenyl rings (C21–C26 and C51–C56) subtend dihedral angles of 88.81 (8), 86.0 (1) and 84.35 (9), 86.4 (1)°, respectively, with the di­hydro­pyridine ring mean plane. These data are in agreement with structural features observed in similar compounds bearing two phenyl rings at the central di­hydro­pyridine ring (Sharma *et al.* 2021 ▸; Zhao *et al.* 2008 ▸; Banerjee *et al.*, 2014 ▸). The conformations of the two mol­ecules are reinforced by intra­molecular C8—H8⋯F2 and C38—H38⋯F4 hydrogen bonds with notably short H⋯F distances (Table 1 ▸). Each mol­ecule also features an intra­molecular C—H⋯π inter­action. The extended structure of (**I**) is consolidated by C—H⋯π and C—F⋯π inter­actions (Table 1 ▸, Fig. 3 ▸).

## Synthesis and crystallization

2-(Tri­fluoro­meth­yl)benzaldehide (1 mol), 1,3-cyclo­hexa­dione (2 mol), aniline (1 mol) and nicotinic acid (10 mol %) were placed in a round-bottom flask and mixed thoroughly with a glass rod before initiating the reaction. Rectified spirit (4 ml) was then added to it, and the reaction mixture was heated to reflux for about 10 h at 80°C with a CaCl_2_ guard tube over the condenser. The completion of the reaction was monitored by TLC. After completion and solvent evaporation, the resulting white precipitate was filtered off. Suitable single crystals were obtained by slow evaporation of an ethano­lic solution of the purified product: m.p. 245–247°C; IR (KBr) 2922, 2866, 1635, 1569, 1492, 1359, 1181 cm^−1^.

^1^H NMR (400 MHz, CDCl_3_): δ (p.p.m.) = 7.71 (*d*, *J* = 8 Hz, 1H, Ar—H), 7.56–7.53 (*m*, 4H, Ar—H), 7.45–7.39 (*m*, 2H, Ar—H), 7.26–7.20 (*m*, 2H, Ar—H), 5.72 (*s*, 1H, condensing carbon *ali*-H), 2.26–2.07 (*m*, 4H, *ali*-CH_2_), 2.06–2.00 (*m*, 4H *ali*-CH_2_), 1.86–1.773 (*m*, 4H, *ali*-CH_2_). ^13^C NMR (400 MHz, CDCl_3_): δ (p.p.m.) = 196.1 (C=O), 151.7 (C—Cl), 144.7, 139.2, 133.1, 130.9, 130.2, 129.9, 129.8, 129.4, 129.2, 126.5, 126.1, 115.8, 100.6, 36.6, 33.7, 28.6, 21.0, 19.4; HRMS (ESI): calculated for C_26_H_22_NO_2_F_3_: 437.1603, found [*m*/*z*, *M* + H]^+^ 438.1602.

## Refinement

Crystal data, data collection and structure refinement details are summarized in Table 2 ▸. The data were treated with the Squeeze program (Spek, 2015 ▸) to take into account a region of disordered electron density of 110.5 Å^3^ (5.1% of the unit-cell volume).

## Supplementary Material

Crystal structure: contains datablock(s) I, General. DOI: 10.1107/S2414314626008254/hb4566sup1.cif

Structure factors: contains datablock(s) I. DOI: 10.1107/S2414314626008254/hb4566Isup2.hkl

Supporting information file. DOI: 10.1107/S2414314626008254/hb4566Isup3.cml

CCDC reference: 2579961

Additional supporting information:  crystallographic information; 3D view; checkCIF report

## Figures and Tables

**Figure 2:**
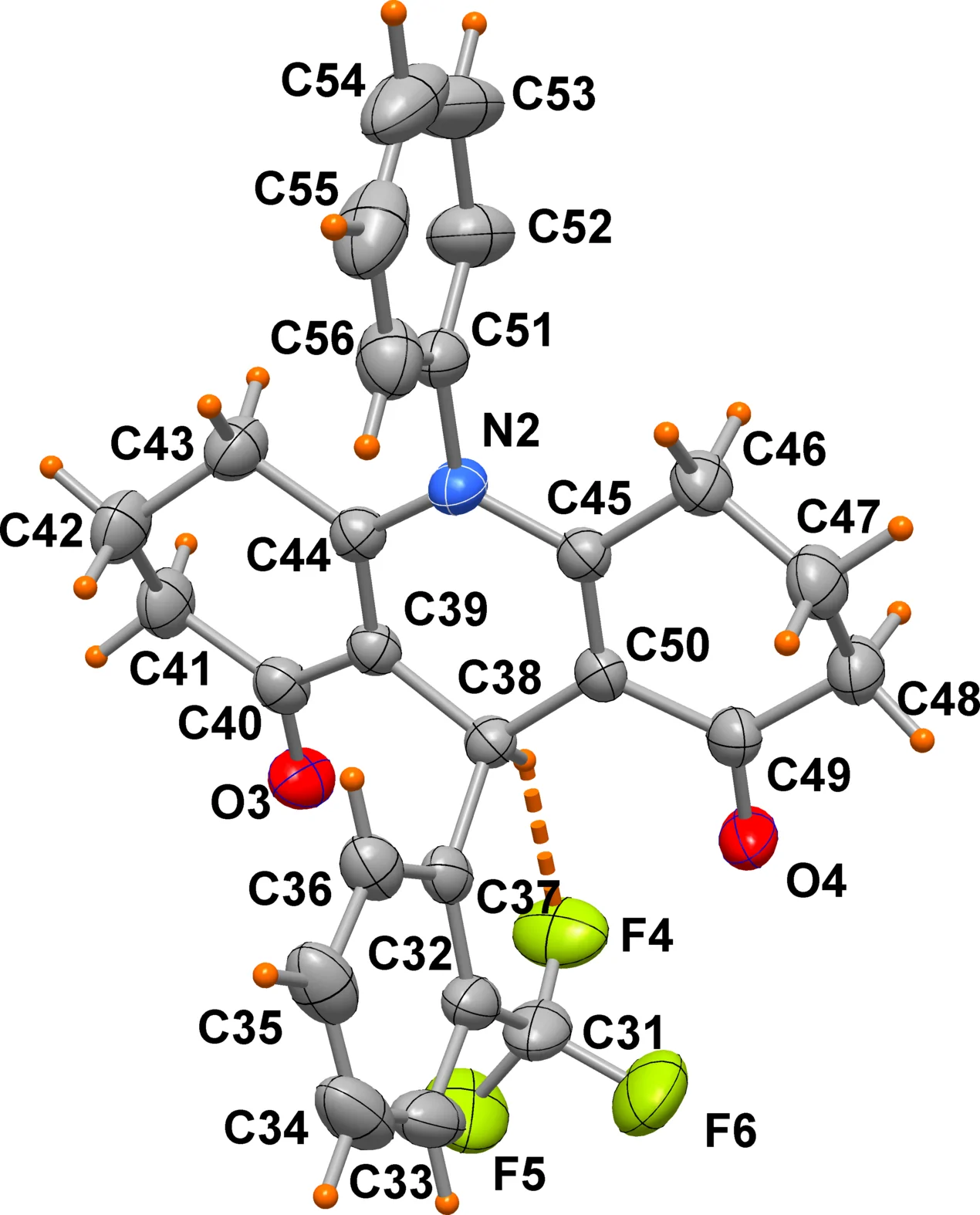
The mol­ecular structure of the second mol­ecule with displacement ellipsoids drawn at the 40% probability level.

**Table 1:** Hydrogen-bond geometry (Å, °) *Cg*, *Cg*5 and *Cg*13 are the centroids of the C2–C7, C21–C26 and C51–C56 rings, respectively.

*D*—H⋯*A*	*D*—H	H⋯*A*	*D*⋯*A*	*D*—H⋯*A*
C8—H8⋯F2	0.98	2.12	2.892 (4)	134
C38—H38⋯F4	0.98	2.15	2.908 (4)	133
C11—H11*B*⋯*Cg*2^i^	0.97	2.87	3.577 (5)	130
C13—H13*B*⋯*Cg*5	0.97	3.00	3.643 (4)	125
C46—H46*B*⋯*Cg*13	0.97	2.98	3.615 (4)	124
C1—F3⋯*Cg*13^ii^	1.33 (1)	3.93 (1)	4.949 (5)	134 (1)

Symmetry codes: (i) 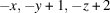; (ii) 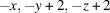.

**Table 2:** Experimental details

Crystal data	
Chemical formula	C_26_H_22_F_3_NO_2_
*M*_r_	437.44
Crystal system, space group	Triclinic, *P* 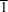
Temperature (K)	293
*a*, *b*, *c* (Å)	9.969 (4), 12.972 (5), 18.577 (7)
α, β, γ (°)	75.400 (8), 76.640 (9), 72.178 (7)
*V* (Å^3^)	2182.1 (15)
*Z*	4
Radiation type	Mo *K*α
μ (mm^−1^)	0.10
Crystal size (mm)	0.25 × 0.24 × 0.24

Data collection	
Diffractometer	Bruker APEXII CCD
Absorption correction	Multi-scan (*SADABS*; Krause *et al.*, 2015 ▸)
*T*_min_, *T*_max_	0.681, 0.745
No. of measured, independent and observed [*I* > 2σ(*I*)] reflections	19525, 6582, 4643
*R*_int_	0.043
θ_max_ (°)	24.1
(sin θ/λ)_max_ (Å^−1^)	0.575

Refinement	
*R*[*F*^2^ > 2σ(*F*^2^)], *wR*(*F*^2^), *S*	0.060, 0.155, 1.05
No. of reflections	6582
No. of parameters	578
H-atom treatment	H-atom parameters constrained
Δρ_max_, Δρ_min_ (e Å^−3^)	0.65, −0.28

Computer programs: *SMART* and, *SAINT* V8.40B (Bruker, 2012 ▸), *SHELXT2014/5* (Sheldrick, 2015*a* ▸), *SHELXL2019/2* (Sheldrick, 2015*b* ▸), *DIAMOND* (Brandenburg, 1999 ▸) and *WinGX* (Farrugia, 2012 ▸).

## full crystallographic data

### 10-Phenyl-9-[2-(trifluoromethyl)phenyl]-3,4,6,7,9,10-hexahydroacridine-1,8(2H,5H)-dione Crystal data

**Table:**

C_26_H_22_F_3_NO_2_	*Z* = 4
*M**_r_* = 437.44	*F*(000) = 912
Triclinic, *P*1	*D*_x_ = 1.332 Mg m^−^^3^
*a* = 9.969 (4) Å	Mo *K*α radiation, λ = 0.71073 Å
*b* = 12.972 (5) Å	Cell parameters from 9125 reflections
*c* = 18.577 (7) Å	θ = 2.2–22.9°
α = 75.400 (8)°	µ = 0.10 mm^−^^1^
β = 76.640 (9)°	*T* = 293 K
γ = 72.178 (7)°	Block, colorless
*V* = 2182.1 (15) Å^3^	0.25 × 0.24 × 0.24 mm

### 10-Phenyl-9-[2-(trifluoromethyl)phenyl]-3,4,6,7,9,10-hexahydroacridine-1,8(2H,5H)-dione Data collection

**Table:**

Bruker APEXII CCD diffractometer	4643 reflections with *I* > 2σ(*I*)
φ and ω scans	*R*_int_ = 0.043
Absorption correction: multi-scan (SADABS; Krause *et al.*, 2015)	θ_max_ = 24.1°, θ_min_ = 2.2°
*T*_min_ = 0.681, *T*_max_ = 0.745	*h* = −11→11
19525 measured reflections	*k* = −14→14
6582 independent reflections	*l* = −21→21

### 10-Phenyl-9-[2-(trifluoromethyl)phenyl]-3,4,6,7,9,10-hexahydroacridine-1,8(2H,5H)-dione Refinement

**Table:**

Refinement on *F*^2^	Hydrogen site location: inferred from neighbouring sites
Least-squares matrix: full	H-atom parameters constrained
*R*[*F*^2^ > 2σ(*F*^2^)] = 0.060	*w* = 1/[σ^2^(*F*_o_^2^) + (0.0562*P*)^2^ + 1.3956*P*] where *P* = (*F*_o_^2^ + 2*F*_c_^2^)/3
*wR*(*F*^2^) = 0.155	(Δ/σ)_max_ < 0.001
*S* = 1.05	Δρ_max_ = 0.65 e Å^−^^3^
6582 reflections	Δρ_min_ = −0.28 e Å^−^^3^
578 parameters	Extinction correction: SHELXL-2019/2 (Sheldrick 2015b), Fc^*^=kFc[1+0.001xFc^2^λ^3^/sin(2θ)]^-1/4^
0 restraints	Extinction coefficient: 0.026 (3)
Primary atom site location: dual	

### 10-Phenyl-9-[2-(trifluoromethyl)phenyl]-3,4,6,7,9,10-hexahydroacridine-1,8(2H,5H)-dione Special details

**Table:**

Geometry. All esds (except the esd in the dihedral angle between two l.s. planes) are estimated using the full covariance matrix. The cell esds are taken into account individually in the estimation of esds in distances, angles and torsion angles; correlations between esds in cell parameters are only used when they are defined by crystal symmetry. An approximate (isotropic) treatment of cell esds is used for estimating esds involving l.s. planes.

### 10-Phenyl-9-[2-(trifluoromethyl)phenyl]-3,4,6,7,9,10-hexahydroacridine-1,8(2H,5H)-dione Fractional atomic coordinates and isotropic or equivalent isotropic displacement parameters (Å^2^)

**Table:**

	*x*	*y*	*z*	*U*_iso_*/*U*_eq_	
F1	0.2204 (4)	0.8231 (2)	1.10557 (15)	0.1292 (11)	
F2	0.3017 (3)	0.6548 (2)	1.10334 (11)	0.0967 (8)	
F3	0.0872 (3)	0.7165 (3)	1.15099 (13)	0.1315 (11)	
N1	0.4441 (3)	0.5337 (2)	0.81358 (12)	0.0489 (6)	
O1	0.1806 (2)	0.48860 (17)	1.06044 (12)	0.0648 (6)	
O2	0.4728 (3)	0.7663 (2)	0.96451 (14)	0.0782 (8)	
C1	0.1880 (5)	0.7379 (3)	1.0928 (2)	0.0702 (10)	
C2	0.1348 (3)	0.7649 (2)	1.01964 (17)	0.0495 (8)	
C3	0.0098 (4)	0.8507 (3)	1.0157 (2)	0.0661 (10)	
H3	−0.031370	0.884869	1.056919	0.079*	
C4	−0.0536 (4)	0.8857 (3)	0.9527 (2)	0.0719 (11)	
H4	−0.136430	0.943369	0.951035	0.086*	
C5	0.0063 (4)	0.8348 (3)	0.8918 (2)	0.0633 (9)	
H5	−0.036311	0.857149	0.848819	0.076*	
C6	0.1296 (3)	0.7508 (2)	0.89520 (17)	0.0491 (8)	
H6	0.169604	0.717460	0.853546	0.059*	
C7	0.1975 (3)	0.7130 (2)	0.95816 (15)	0.0414 (7)	
C8	0.3348 (3)	0.6185 (2)	0.95213 (15)	0.0395 (7)	
H8	0.370246	0.597239	1.000204	0.047*	
C9	0.3038 (3)	0.5189 (2)	0.93716 (15)	0.0402 (7)	
C10	0.2187 (3)	0.4603 (2)	0.99901 (16)	0.0475 (7)	
C11	0.1793 (4)	0.3647 (3)	0.98618 (18)	0.0627 (9)	
H11A	0.243201	0.296855	1.007982	0.075*	
H11B	0.083158	0.365410	1.012759	0.075*	
C12	0.1853 (5)	0.3640 (4)	0.9072 (2)	0.0945 (14)	
H12A	0.103365	0.419375	0.889238	0.113*	
H12B	0.178438	0.292573	0.903407	0.113*	
C13	0.3195 (4)	0.3862 (3)	0.85702 (18)	0.0608 (9)	
H13A	0.398736	0.321345	0.865595	0.073*	
H13B	0.308126	0.399745	0.804700	0.073*	
C14	0.3536 (3)	0.4837 (2)	0.87122 (16)	0.0441 (7)	
C15	0.4959 (3)	0.6155 (2)	0.82537 (15)	0.0438 (7)	
C16	0.6094 (4)	0.6529 (3)	0.76543 (18)	0.0609 (9)	
H16A	0.700963	0.600121	0.771352	0.073*	
H16B	0.589487	0.654807	0.716338	0.073*	
C17	0.6181 (4)	0.7647 (3)	0.7685 (2)	0.0710 (10)	
H17A	0.533302	0.819859	0.753626	0.085*	
H17B	0.700540	0.781604	0.733339	0.085*	
C18	0.6303 (4)	0.7692 (3)	0.8468 (2)	0.0690 (10)	
H18A	0.721929	0.721913	0.858691	0.083*	
H18B	0.626604	0.844120	0.848619	0.083*	
C19	0.5128 (3)	0.7329 (3)	0.90464 (19)	0.0547 (8)	
C20	0.4490 (3)	0.6548 (2)	0.89036 (16)	0.0422 (7)	
C21	0.4833 (3)	0.5019 (2)	0.74031 (16)	0.0492 (8)	
C22	0.6084 (4)	0.4239 (3)	0.72341 (19)	0.0749 (11)	
H22	0.669887	0.391041	0.758403	0.090*	
C23	0.6413 (5)	0.3949 (3)	0.6533 (2)	0.0952 (15)	
H23	0.726553	0.343059	0.640795	0.114*	
C24	0.5507 (6)	0.4412 (3)	0.6028 (2)	0.0925 (14)	
H24	0.573499	0.420225	0.556170	0.111*	
C25	0.4265 (5)	0.5183 (3)	0.6198 (2)	0.0825 (12)	
H25	0.364343	0.549658	0.585005	0.099*	
C26	0.3931 (4)	0.5497 (3)	0.68884 (18)	0.0617 (9)	
H26	0.309232	0.603298	0.700312	0.074*	
F4	−0.0632 (3)	0.7921 (2)	0.48429 (13)	0.0967 (8)	
F5	−0.1704 (3)	0.6662 (2)	0.52946 (15)	0.1129 (9)	
F6	0.0513 (3)	0.6246 (2)	0.48690 (15)	0.1134 (9)	
N2	0.1403 (2)	0.99396 (19)	0.66515 (13)	0.0478 (6)	
O3	−0.2618 (2)	0.94173 (18)	0.60037 (13)	0.0618 (6)	
O4	0.2417 (2)	0.76129 (19)	0.48984 (13)	0.0652 (7)	
C31	−0.0484 (4)	0.6930 (4)	0.5266 (2)	0.0756 (11)	
C32	−0.0190 (3)	0.6775 (3)	0.60499 (19)	0.0550 (8)	
C33	−0.0269 (4)	0.5748 (3)	0.6506 (3)	0.0782 (12)	
H33	−0.045087	0.522883	0.630315	0.094*	
C34	−0.0085 (4)	0.5492 (3)	0.7242 (3)	0.0835 (12)	
H34	−0.013076	0.480208	0.753191	0.100*	
C35	0.0169 (4)	0.6256 (3)	0.7554 (2)	0.0704 (10)	
H35	0.025444	0.610163	0.806052	0.085*	
C36	0.0293 (3)	0.7248 (3)	0.71040 (18)	0.0537 (8)	
H36	0.049639	0.775147	0.731395	0.064*	
C37	0.0129 (3)	0.7543 (2)	0.63458 (17)	0.0441 (7)	
C38	0.0324 (3)	0.8687 (2)	0.59299 (15)	0.0399 (7)	
H38	0.002869	0.885420	0.543620	0.048*	
C39	−0.0600 (3)	0.9568 (2)	0.63675 (15)	0.0398 (7)	
C40	−0.2142 (3)	0.9848 (2)	0.63687 (17)	0.0476 (7)	
C41	−0.3121 (3)	1.0686 (3)	0.68136 (19)	0.0622 (9)	
H41A	−0.402351	1.049626	0.700830	0.075*	
H41B	−0.331163	1.140509	0.648226	0.075*	
C42	−0.2494 (3)	1.0751 (3)	0.74656 (19)	0.0648 (9)	
H42A	−0.310892	1.135589	0.770286	0.078*	
H42B	−0.244333	1.007219	0.783946	0.078*	
C43	−0.1014 (3)	1.0927 (3)	0.71922 (19)	0.0587 (9)	
H43A	−0.109026	1.166939	0.689723	0.070*	
H43B	−0.057935	1.086036	0.762403	0.070*	
C44	−0.0069 (3)	1.0110 (2)	0.67190 (16)	0.0437 (7)	
C45	0.2350 (3)	0.9284 (2)	0.61572 (15)	0.0413 (7)	
C46	0.3890 (3)	0.9300 (3)	0.60123 (18)	0.0560 (8)	
H46A	0.400612	0.996362	0.565023	0.067*	
H46B	0.414826	0.932677	0.647825	0.067*	
C47	0.4882 (3)	0.8312 (3)	0.57170 (19)	0.0618 (9)	
H47A	0.492204	0.766221	0.611630	0.074*	
H47B	0.583688	0.841821	0.555942	0.074*	
C48	0.4396 (3)	0.8134 (3)	0.50599 (19)	0.0601 (9)	
H48A	0.452009	0.872295	0.463048	0.072*	
H48B	0.498725	0.744206	0.492174	0.072*	
C49	0.2854 (3)	0.8106 (2)	0.52383 (16)	0.0483 (8)	
C50	0.1878 (3)	0.8700 (2)	0.58075 (15)	0.0402 (7)	
C51	0.1971 (3)	1.0489 (3)	0.70523 (18)	0.0517 (8)	
C52	0.2128 (4)	1.1532 (3)	0.6748 (2)	0.0803 (11)	
H52	0.185249	1.190256	0.628514	0.096*	
C53	0.2709 (5)	1.2035 (4)	0.7143 (4)	0.1043 (16)	
H53	0.281921	1.274340	0.694669	0.125*	
C54	0.3112 (5)	1.1472 (5)	0.7820 (4)	0.1053 (18)	
H54	0.350656	1.180168	0.807978	0.126*	
C55	0.2950 (4)	1.0449 (5)	0.8118 (2)	0.0917 (14)	
H55	0.322057	1.008363	0.858265	0.110*	
C56	0.2385 (4)	0.9944 (3)	0.77389 (19)	0.0673 (10)	
H56	0.228194	0.923520	0.794425	0.081*	

### 10-Phenyl-9-[2-(trifluoromethyl)phenyl]-3,4,6,7,9,10-hexahydroacridine-1,8(2H,5H)-dione Atomic displacement parameters (Å^2^)

**Table:**

	*U*^11^	*U*^22^	*U*^33^	*U*^12^	*U*^13^	*U*^23^
F1	0.199 (3)	0.1068 (19)	0.118 (2)	−0.046 (2)	−0.056 (2)	−0.0519 (17)
F2	0.1153 (19)	0.1105 (18)	0.0583 (13)	0.0051 (16)	−0.0314 (13)	−0.0333 (13)
F3	0.117 (2)	0.212 (3)	0.0537 (14)	−0.052 (2)	0.0155 (14)	−0.0224 (16)
N1	0.0548 (15)	0.0566 (15)	0.0376 (13)	−0.0233 (13)	0.0065 (12)	−0.0155 (12)
O1	0.0820 (16)	0.0618 (14)	0.0453 (13)	−0.0276 (12)	0.0131 (12)	−0.0119 (11)
O2	0.0857 (18)	0.0885 (18)	0.0807 (17)	−0.0467 (15)	0.0055 (14)	−0.0410 (15)
C1	0.088 (3)	0.076 (3)	0.053 (2)	−0.025 (2)	0.003 (2)	−0.031 (2)
C2	0.0526 (19)	0.0447 (17)	0.0516 (19)	−0.0160 (16)	0.0029 (15)	−0.0169 (15)
C3	0.063 (2)	0.0472 (19)	0.081 (3)	−0.0106 (18)	0.011 (2)	−0.0256 (19)
C4	0.052 (2)	0.046 (2)	0.102 (3)	−0.0075 (17)	−0.002 (2)	−0.005 (2)
C5	0.056 (2)	0.056 (2)	0.069 (2)	−0.0123 (18)	−0.0124 (19)	0.0037 (18)
C6	0.0465 (19)	0.0472 (18)	0.0473 (18)	−0.0109 (15)	−0.0050 (15)	−0.0025 (14)
C7	0.0424 (17)	0.0377 (15)	0.0430 (17)	−0.0155 (13)	0.0004 (14)	−0.0068 (13)
C8	0.0414 (16)	0.0393 (15)	0.0361 (15)	−0.0105 (13)	−0.0037 (13)	−0.0068 (12)
C9	0.0406 (16)	0.0358 (15)	0.0398 (16)	−0.0086 (13)	0.0008 (13)	−0.0082 (13)
C10	0.0500 (18)	0.0423 (17)	0.0443 (18)	−0.0129 (14)	0.0016 (15)	−0.0057 (14)
C11	0.071 (2)	0.057 (2)	0.062 (2)	−0.0309 (18)	0.0045 (18)	−0.0132 (17)
C12	0.128 (4)	0.096 (3)	0.081 (3)	−0.074 (3)	0.012 (3)	−0.028 (2)
C13	0.072 (2)	0.058 (2)	0.058 (2)	−0.0301 (18)	0.0084 (18)	−0.0241 (16)
C14	0.0442 (17)	0.0408 (16)	0.0442 (17)	−0.0115 (14)	0.0005 (14)	−0.0097 (14)
C15	0.0393 (17)	0.0481 (17)	0.0407 (16)	−0.0133 (14)	−0.0011 (13)	−0.0058 (14)
C16	0.055 (2)	0.076 (2)	0.0506 (19)	−0.0309 (18)	0.0046 (16)	−0.0059 (17)
C17	0.064 (2)	0.076 (2)	0.069 (2)	−0.0351 (19)	−0.0009 (19)	0.0046 (19)
C18	0.066 (2)	0.070 (2)	0.078 (2)	−0.0375 (19)	−0.008 (2)	−0.0070 (19)
C19	0.0487 (19)	0.0530 (19)	0.064 (2)	−0.0170 (16)	−0.0071 (17)	−0.0115 (17)
C20	0.0387 (16)	0.0415 (16)	0.0454 (17)	−0.0129 (13)	−0.0050 (14)	−0.0056 (13)
C21	0.0537 (19)	0.0498 (18)	0.0377 (16)	−0.0093 (15)	0.0007 (16)	−0.0104 (14)
C22	0.077 (3)	0.071 (2)	0.051 (2)	0.014 (2)	−0.0060 (19)	−0.0120 (18)
C23	0.118 (4)	0.073 (3)	0.054 (2)	0.025 (2)	0.005 (3)	−0.020 (2)
C24	0.147 (4)	0.071 (3)	0.044 (2)	−0.007 (3)	−0.003 (3)	−0.022 (2)
C25	0.106 (3)	0.089 (3)	0.053 (2)	−0.016 (3)	−0.022 (2)	−0.019 (2)
C26	0.058 (2)	0.066 (2)	0.056 (2)	−0.0057 (17)	−0.0099 (18)	−0.0174 (18)
F4	0.139 (2)	0.1017 (18)	0.0788 (15)	−0.0508 (16)	−0.0457 (15)	−0.0198 (14)
F5	0.0898 (17)	0.157 (2)	0.131 (2)	−0.0670 (17)	−0.0164 (15)	−0.0573 (18)
F6	0.1114 (19)	0.131 (2)	0.1162 (19)	−0.0323 (16)	0.0100 (16)	−0.0832 (17)
N2	0.0445 (15)	0.0555 (15)	0.0523 (15)	−0.0152 (12)	−0.0076 (12)	−0.0241 (13)
O3	0.0479 (13)	0.0669 (14)	0.0786 (16)	−0.0159 (11)	−0.0175 (12)	−0.0211 (12)
O4	0.0586 (14)	0.0834 (16)	0.0686 (15)	−0.0318 (12)	0.0076 (12)	−0.0418 (13)
C31	0.067 (2)	0.084 (3)	0.098 (3)	−0.035 (2)	−0.001 (2)	−0.049 (3)
C32	0.0462 (19)	0.0500 (19)	0.073 (2)	−0.0180 (15)	0.0001 (17)	−0.0215 (17)
C33	0.064 (2)	0.054 (2)	0.120 (4)	−0.0273 (19)	0.001 (2)	−0.023 (2)
C34	0.064 (3)	0.055 (2)	0.112 (4)	−0.0199 (19)	−0.001 (3)	0.011 (2)
C35	0.056 (2)	0.063 (2)	0.073 (2)	−0.0122 (18)	−0.0076 (19)	0.013 (2)
C36	0.0457 (18)	0.0515 (19)	0.058 (2)	−0.0125 (15)	−0.0066 (16)	−0.0017 (16)
C37	0.0310 (16)	0.0436 (17)	0.0543 (19)	−0.0086 (13)	−0.0015 (14)	−0.0100 (14)
C38	0.0423 (17)	0.0438 (16)	0.0365 (15)	−0.0143 (13)	−0.0052 (13)	−0.0104 (13)
C39	0.0368 (16)	0.0398 (15)	0.0429 (16)	−0.0108 (13)	−0.0082 (13)	−0.0062 (13)
C40	0.0459 (19)	0.0460 (17)	0.0492 (18)	−0.0137 (15)	−0.0086 (15)	−0.0040 (15)
C41	0.0420 (18)	0.070 (2)	0.070 (2)	−0.0071 (16)	−0.0038 (17)	−0.0201 (18)
C42	0.052 (2)	0.072 (2)	0.067 (2)	−0.0034 (17)	−0.0043 (18)	−0.0277 (19)
C43	0.051 (2)	0.062 (2)	0.066 (2)	−0.0077 (16)	−0.0100 (17)	−0.0267 (17)
C44	0.0433 (18)	0.0454 (17)	0.0438 (16)	−0.0124 (14)	−0.0059 (14)	−0.0115 (14)
C45	0.0408 (17)	0.0449 (16)	0.0402 (16)	−0.0150 (14)	−0.0043 (13)	−0.0096 (13)
C46	0.0436 (18)	0.070 (2)	0.062 (2)	−0.0206 (16)	−0.0051 (16)	−0.0226 (17)
C47	0.0390 (18)	0.077 (2)	0.072 (2)	−0.0146 (17)	−0.0047 (17)	−0.0226 (19)
C48	0.0453 (19)	0.075 (2)	0.064 (2)	−0.0187 (17)	0.0028 (16)	−0.0284 (18)
C49	0.0493 (19)	0.0535 (18)	0.0431 (17)	−0.0183 (15)	0.0001 (15)	−0.0126 (15)
C50	0.0389 (16)	0.0420 (16)	0.0386 (15)	−0.0133 (13)	−0.0022 (13)	−0.0067 (13)
C51	0.0447 (18)	0.058 (2)	0.060 (2)	−0.0171 (15)	−0.0025 (16)	−0.0250 (17)
C52	0.086 (3)	0.062 (2)	0.105 (3)	−0.025 (2)	−0.024 (2)	−0.024 (2)
C53	0.090 (3)	0.073 (3)	0.171 (5)	−0.026 (3)	−0.023 (4)	−0.054 (3)
C54	0.068 (3)	0.134 (5)	0.146 (5)	−0.016 (3)	−0.014 (3)	−0.101 (4)
C55	0.066 (3)	0.150 (5)	0.078 (3)	−0.022 (3)	−0.012 (2)	−0.064 (3)
C56	0.060 (2)	0.092 (3)	0.054 (2)	−0.023 (2)	−0.0037 (18)	−0.024 (2)

### 10-Phenyl-9-[2-(trifluoromethyl)phenyl]-3,4,6,7,9,10-hexahydroacridine-1,8(2H,5H)-dione Geometric parameters (Å, º)

**Table:**

F1—C1	1.329 (4)	F4—C31	1.312 (5)
F2—C1	1.316 (4)	F5—C31	1.350 (4)
F3—C1	1.332 (4)	F6—C31	1.331 (4)
N1—C15	1.394 (4)	N2—C44	1.396 (4)
N1—C14	1.394 (3)	N2—C45	1.400 (3)
N1—C21	1.454 (3)	N2—C51	1.447 (4)
O1—C10	1.228 (3)	O3—C40	1.224 (3)
O2—C19	1.231 (4)	O4—C49	1.224 (3)
C1—C2	1.494 (5)	C31—C32	1.503 (5)
C2—C3	1.395 (4)	C32—C37	1.396 (4)
C2—C7	1.396 (4)	C32—C33	1.401 (5)
C3—C4	1.370 (5)	C33—C34	1.364 (6)
C3—H3	0.9300	C33—H33	0.9300
C4—C5	1.377 (5)	C34—C35	1.375 (5)
C4—H4	0.9300	C34—H34	0.9300
C5—C6	1.373 (4)	C35—C36	1.371 (4)
C5—H5	0.9300	C35—H35	0.9300
C6—C7	1.393 (4)	C36—C37	1.397 (4)
C6—H6	0.9300	C36—H36	0.9300
C7—C8	1.537 (4)	C37—C38	1.539 (4)
C8—C20	1.512 (4)	C38—C39	1.514 (4)
C8—C9	1.519 (4)	C38—C50	1.518 (4)
C8—H8	0.9800	C38—H38	0.9800
C9—C14	1.348 (4)	C39—C44	1.349 (4)
C9—C10	1.457 (4)	C39—C40	1.467 (4)
C10—C11	1.496 (4)	C40—C41	1.500 (4)
C11—C12	1.457 (5)	C41—C42	1.516 (4)
C11—H11A	0.9700	C41—H41A	0.9700
C11—H11B	0.9700	C41—H41B	0.9700
C12—C13	1.505 (5)	C42—C43	1.513 (4)
C12—H12A	0.9700	C42—H42A	0.9700
C12—H12B	0.9700	C42—H42B	0.9700
C13—C14	1.503 (4)	C43—C44	1.503 (4)
C13—H13A	0.9700	C43—H43A	0.9700
C13—H13B	0.9700	C43—H43B	0.9700
C15—C20	1.356 (4)	C45—C50	1.350 (4)
C15—C16	1.496 (4)	C45—C46	1.502 (4)
C16—C17	1.495 (4)	C46—C47	1.499 (4)
C16—H16A	0.9700	C46—H46A	0.9700
C16—H16B	0.9700	C46—H46B	0.9700
C17—C18	1.504 (5)	C47—C48	1.505 (4)
C17—H17A	0.9700	C47—H47A	0.9700
C17—H17B	0.9700	C47—H47B	0.9700
C18—C19	1.500 (4)	C48—C49	1.505 (4)
C18—H18A	0.9700	C48—H48A	0.9700
C18—H18B	0.9700	C48—H48B	0.9700
C19—C20	1.452 (4)	C49—C50	1.461 (4)
C21—C26	1.366 (4)	C51—C52	1.371 (5)
C21—C22	1.372 (4)	C51—C56	1.381 (5)
C22—C23	1.386 (5)	C52—C53	1.404 (6)
C22—H22	0.9300	C52—H52	0.9300
C23—C24	1.354 (6)	C53—C54	1.366 (7)
C23—H23	0.9300	C53—H53	0.9300
C24—C25	1.362 (6)	C54—C55	1.347 (7)
C24—H24	0.9300	C54—H54	0.9300
C25—C26	1.380 (4)	C55—C56	1.372 (5)
C25—H25	0.9300	C55—H55	0.9300
C26—H26	0.9300	C56—H56	0.9300
			
C15—N1—C14	120.4 (2)	C44—N2—C45	120.2 (2)
C15—N1—C21	119.6 (2)	C44—N2—C51	120.8 (2)
C14—N1—C21	120.0 (2)	C45—N2—C51	118.9 (2)
F2—C1—F1	104.4 (3)	F4—C31—F6	106.4 (3)
F2—C1—F3	105.3 (3)	F4—C31—F5	105.3 (4)
F1—C1—F3	104.1 (3)	F6—C31—F5	104.2 (3)
F2—C1—C2	118.7 (3)	F4—C31—C32	117.6 (3)
F1—C1—C2	111.9 (3)	F6—C31—C32	112.0 (4)
F3—C1—C2	111.3 (3)	F5—C31—C32	110.2 (3)
C3—C2—C7	119.7 (3)	C37—C32—C33	119.3 (3)
C3—C2—C1	114.0 (3)	C37—C32—C31	126.4 (3)
C7—C2—C1	126.3 (3)	C33—C32—C31	114.3 (3)
C4—C3—C2	121.5 (3)	C34—C33—C32	121.6 (4)
C4—C3—H3	119.2	C34—C33—H33	119.2
C2—C3—H3	119.2	C32—C33—H33	119.2
C3—C4—C5	119.5 (3)	C33—C34—C35	119.9 (4)
C3—C4—H4	120.2	C33—C34—H34	120.0
C5—C4—H4	120.2	C35—C34—H34	120.0
C6—C5—C4	119.2 (3)	C36—C35—C34	118.7 (4)
C6—C5—H5	120.4	C36—C35—H35	120.6
C4—C5—H5	120.4	C34—C35—H35	120.6
C5—C6—C7	123.1 (3)	C35—C36—C37	123.3 (3)
C5—C6—H6	118.5	C35—C36—H36	118.3
C7—C6—H6	118.5	C37—C36—H36	118.3
C6—C7—C2	117.0 (3)	C32—C37—C36	116.9 (3)
C6—C7—C8	116.1 (2)	C32—C37—C38	127.2 (3)
C2—C7—C8	126.9 (3)	C36—C37—C38	115.8 (3)
C20—C8—C9	110.0 (2)	C39—C38—C50	109.8 (2)
C20—C8—C7	110.9 (2)	C39—C38—C37	110.6 (2)
C9—C8—C7	110.3 (2)	C50—C38—C37	110.8 (2)
C20—C8—H8	108.5	C39—C38—H38	108.5
C9—C8—H8	108.5	C50—C38—H38	108.5
C7—C8—H8	108.5	C37—C38—H38	108.5
C14—C9—C10	120.7 (3)	C44—C39—C40	120.4 (3)
C14—C9—C8	122.8 (2)	C44—C39—C38	123.4 (3)
C10—C9—C8	116.4 (2)	C40—C39—C38	116.2 (2)
O1—C10—C9	120.6 (3)	O3—C40—C39	120.6 (3)
O1—C10—C11	120.8 (3)	O3—C40—C41	120.7 (3)
C9—C10—C11	118.6 (3)	C39—C40—C41	118.8 (3)
C12—C11—C10	114.6 (3)	C40—C41—C42	112.3 (3)
C12—C11—H11A	108.6	C40—C41—H41A	109.2
C10—C11—H11A	108.6	C42—C41—H41A	109.2
C12—C11—H11B	108.6	C40—C41—H41B	109.2
C10—C11—H11B	108.6	C42—C41—H41B	109.2
H11A—C11—H11B	107.6	H41A—C41—H41B	107.9
C11—C12—C13	113.5 (3)	C43—C42—C41	110.6 (3)
C11—C12—H12A	108.9	C43—C42—H42A	109.5
C13—C12—H12A	108.9	C41—C42—H42A	109.5
C11—C12—H12B	108.9	C43—C42—H42B	109.5
C13—C12—H12B	108.9	C41—C42—H42B	109.5
H12A—C12—H12B	107.7	H42A—C42—H42B	108.1
C14—C13—C12	112.1 (3)	C44—C43—C42	112.1 (3)
C14—C13—H13A	109.2	C44—C43—H43A	109.2
C12—C13—H13A	109.2	C42—C43—H43A	109.2
C14—C13—H13B	109.2	C44—C43—H43B	109.2
C12—C13—H13B	109.2	C42—C43—H43B	109.2
H13A—C13—H13B	107.9	H43A—C43—H43B	107.9
C9—C14—N1	121.4 (2)	C39—C44—N2	120.9 (2)
C9—C14—C13	122.0 (3)	C39—C44—C43	122.4 (3)
N1—C14—C13	116.5 (2)	N2—C44—C43	116.7 (2)
C20—C15—N1	120.7 (2)	C50—C45—N2	121.0 (2)
C20—C15—C16	122.1 (3)	C50—C45—C46	122.0 (2)
N1—C15—C16	117.2 (2)	N2—C45—C46	117.0 (2)
C17—C16—C15	112.2 (3)	C47—C46—C45	112.8 (3)
C17—C16—H16A	109.2	C47—C46—H46A	109.0
C15—C16—H16A	109.2	C45—C46—H46A	109.0
C17—C16—H16B	109.2	C47—C46—H46B	109.0
C15—C16—H16B	109.2	C45—C46—H46B	109.0
H16A—C16—H16B	107.9	H46A—C46—H46B	107.8
C16—C17—C18	110.8 (3)	C46—C47—C48	111.2 (3)
C16—C17—H17A	109.5	C46—C47—H47A	109.4
C18—C17—H17A	109.5	C48—C47—H47A	109.4
C16—C17—H17B	109.5	C46—C47—H47B	109.4
C18—C17—H17B	109.5	C48—C47—H47B	109.4
H17A—C17—H17B	108.1	H47A—C47—H47B	108.0
C19—C18—C17	111.7 (3)	C49—C48—C47	112.1 (3)
C19—C18—H18A	109.3	C49—C48—H48A	109.2
C17—C18—H18A	109.3	C47—C48—H48A	109.2
C19—C18—H18B	109.3	C49—C48—H48B	109.2
C17—C18—H18B	109.3	C47—C48—H48B	109.2
H18A—C18—H18B	107.9	H48A—C48—H48B	107.9
O2—C19—C20	120.0 (3)	O4—C49—C50	120.4 (3)
O2—C19—C18	120.8 (3)	O4—C49—C48	120.4 (3)
C20—C19—C18	119.2 (3)	C50—C49—C48	119.1 (3)
C15—C20—C19	120.0 (3)	C45—C50—C49	120.3 (3)
C15—C20—C8	123.4 (2)	C45—C50—C38	123.2 (2)
C19—C20—C8	116.6 (2)	C49—C50—C38	116.4 (2)
C26—C21—C22	120.4 (3)	C52—C51—C56	120.0 (3)
C26—C21—N1	119.1 (3)	C52—C51—N2	120.5 (3)
C22—C21—N1	120.5 (3)	C56—C51—N2	119.5 (3)
C21—C22—C23	118.8 (4)	C51—C52—C53	119.1 (4)
C21—C22—H22	120.6	C51—C52—H52	120.4
C23—C22—H22	120.6	C53—C52—H52	120.4
C24—C23—C22	120.6 (4)	C54—C53—C52	119.4 (4)
C24—C23—H23	119.7	C54—C53—H53	120.3
C22—C23—H23	119.7	C52—C53—H53	120.3
C23—C24—C25	120.4 (3)	C55—C54—C53	121.3 (4)
C23—C24—H24	119.8	C55—C54—H54	119.4
C25—C24—H24	119.8	C53—C54—H54	119.4
C24—C25—C26	119.8 (4)	C54—C55—C56	120.2 (5)
C24—C25—H25	120.1	C54—C55—H55	119.9
C26—C25—H25	120.1	C56—C55—H55	119.9
C21—C26—C25	119.9 (3)	C55—C56—C51	120.0 (4)
C21—C26—H26	120.0	C55—C56—H56	120.0
C25—C26—H26	120.0	C51—C56—H56	120.0
			
F2—C1—C2—C3	177.0 (3)	F4—C31—C32—C37	−9.1 (5)
F1—C1—C2—C3	−61.4 (4)	F6—C31—C32—C37	114.7 (4)
F3—C1—C2—C3	54.6 (4)	F5—C31—C32—C37	−129.7 (3)
F2—C1—C2—C7	−2.8 (5)	F4—C31—C32—C33	170.5 (3)
F1—C1—C2—C7	118.8 (4)	F6—C31—C32—C33	−65.8 (4)
F3—C1—C2—C7	−125.2 (4)	F5—C31—C32—C33	49.8 (4)
C7—C2—C3—C4	0.0 (5)	C37—C32—C33—C34	2.1 (5)
C1—C2—C3—C4	−179.8 (3)	C31—C32—C33—C34	−177.4 (3)
C2—C3—C4—C5	0.5 (5)	C32—C33—C34—C35	0.8 (6)
C3—C4—C5—C6	−0.8 (5)	C33—C34—C35—C36	−2.8 (5)
C4—C5—C6—C7	0.6 (5)	C34—C35—C36—C37	2.1 (5)
C5—C6—C7—C2	−0.1 (4)	C33—C32—C37—C36	−2.7 (4)
C5—C6—C7—C8	−179.8 (3)	C31—C32—C37—C36	176.8 (3)
C3—C2—C7—C6	−0.2 (4)	C33—C32—C37—C38	177.3 (3)
C1—C2—C7—C6	179.6 (3)	C31—C32—C37—C38	−3.2 (5)
C3—C2—C7—C8	179.4 (3)	C35—C36—C37—C32	0.7 (4)
C1—C2—C7—C8	−0.8 (5)	C35—C36—C37—C38	−179.4 (3)
C6—C7—C8—C20	63.2 (3)	C32—C37—C38—C39	128.3 (3)
C2—C7—C8—C20	−116.4 (3)	C36—C37—C38—C39	−51.6 (3)
C6—C7—C8—C9	−58.9 (3)	C32—C37—C38—C50	−109.6 (3)
C2—C7—C8—C9	121.4 (3)	C36—C37—C38—C50	70.4 (3)
C20—C8—C9—C14	−11.0 (4)	C50—C38—C39—C44	−12.3 (4)
C7—C8—C9—C14	111.6 (3)	C37—C38—C39—C44	110.3 (3)
C20—C8—C9—C10	167.6 (2)	C50—C38—C39—C40	165.3 (2)
C7—C8—C9—C10	−69.7 (3)	C37—C38—C39—C40	−72.1 (3)
C14—C9—C10—O1	175.7 (3)	C44—C39—C40—O3	174.5 (3)
C8—C9—C10—O1	−3.0 (4)	C38—C39—C40—O3	−3.2 (4)
C14—C9—C10—C11	−4.1 (4)	C44—C39—C40—C41	−4.4 (4)
C8—C9—C10—C11	177.2 (3)	C38—C39—C40—C41	178.0 (3)
O1—C10—C11—C12	159.2 (3)	O3—C40—C41—C42	155.2 (3)
C9—C10—C11—C12	−21.1 (5)	C39—C40—C41—C42	−25.9 (4)
C10—C11—C12—C13	46.6 (5)	C40—C41—C42—C43	52.9 (4)
C11—C12—C13—C14	−47.1 (5)	C41—C42—C43—C44	−50.7 (4)
C10—C9—C14—N1	−175.0 (3)	C40—C39—C44—N2	−172.8 (3)
C8—C9—C14—N1	3.6 (4)	C38—C39—C44—N2	4.7 (4)
C10—C9—C14—C13	2.5 (4)	C40—C39—C44—C43	6.6 (4)
C8—C9—C14—C13	−178.9 (3)	C38—C39—C44—C43	−175.9 (3)
C15—N1—C14—C9	5.8 (4)	C45—N2—C44—C39	6.3 (4)
C21—N1—C14—C9	−173.3 (3)	C51—N2—C44—C39	−177.6 (3)
C15—N1—C14—C13	−171.9 (3)	C45—N2—C44—C43	−173.1 (3)
C21—N1—C14—C13	9.1 (4)	C51—N2—C44—C43	3.0 (4)
C12—C13—C14—C9	22.8 (5)	C42—C43—C44—C39	21.7 (4)
C12—C13—C14—N1	−159.5 (3)	C42—C43—C44—N2	−158.9 (3)
C14—N1—C15—C20	−6.0 (4)	C44—N2—C45—C50	−8.0 (4)
C21—N1—C15—C20	173.0 (3)	C51—N2—C45—C50	175.8 (3)
C14—N1—C15—C16	170.9 (3)	C44—N2—C45—C46	169.5 (3)
C21—N1—C15—C16	−10.1 (4)	C51—N2—C45—C46	−6.7 (4)
C20—C15—C16—C17	−24.4 (4)	C50—C45—C46—C47	−23.0 (4)
N1—C15—C16—C17	158.8 (3)	N2—C45—C46—C47	159.5 (3)
C15—C16—C17—C18	51.7 (4)	C45—C46—C47—C48	50.2 (4)
C16—C17—C18—C19	−53.5 (4)	C46—C47—C48—C49	−51.9 (4)
C17—C18—C19—O2	−153.7 (3)	C47—C48—C49—O4	−154.9 (3)
C17—C18—C19—C20	28.1 (4)	C47—C48—C49—C50	26.8 (4)
N1—C15—C20—C19	174.6 (3)	N2—C45—C50—C49	174.0 (2)
C16—C15—C20—C19	−2.2 (4)	C46—C45—C50—C49	−3.4 (4)
N1—C15—C20—C8	−3.0 (4)	N2—C45—C50—C38	−1.3 (4)
C16—C15—C20—C8	−179.8 (3)	C46—C45—C50—C38	−178.7 (3)
O2—C19—C20—C15	−178.1 (3)	O4—C49—C50—C45	−177.0 (3)
C18—C19—C20—C15	0.1 (5)	C48—C49—C50—C45	1.3 (4)
O2—C19—C20—C8	−0.3 (4)	O4—C49—C50—C38	−1.4 (4)
C18—C19—C20—C8	177.9 (3)	C48—C49—C50—C38	176.9 (3)
C9—C8—C20—C15	10.8 (4)	C39—C38—C50—C45	10.6 (4)
C7—C8—C20—C15	−111.5 (3)	C37—C38—C50—C45	−112.0 (3)
C9—C8—C20—C19	−166.9 (2)	C39—C38—C50—C49	−164.9 (2)
C7—C8—C20—C19	70.8 (3)	C37—C38—C50—C49	72.6 (3)
C15—N1—C21—C26	−95.3 (4)	C44—N2—C51—C52	−85.7 (4)
C14—N1—C21—C26	83.7 (4)	C45—N2—C51—C52	90.5 (4)
C15—N1—C21—C22	85.6 (4)	C44—N2—C51—C56	95.7 (4)
C14—N1—C21—C22	−95.4 (4)	C45—N2—C51—C56	−88.1 (3)
C26—C21—C22—C23	0.2 (6)	C56—C51—C52—C53	−0.1 (5)
N1—C21—C22—C23	179.3 (3)	N2—C51—C52—C53	−178.7 (3)
C21—C22—C23—C24	−1.1 (7)	C51—C52—C53—C54	0.3 (6)
C22—C23—C24—C25	0.9 (7)	C52—C53—C54—C55	−0.6 (7)
C23—C24—C25—C26	0.3 (7)	C53—C54—C55—C56	0.8 (7)
C22—C21—C26—C25	1.0 (5)	C54—C55—C56—C51	−0.6 (6)
N1—C21—C26—C25	−178.2 (3)	C52—C51—C56—C55	0.2 (5)
C24—C25—C26—C21	−1.2 (6)	N2—C51—C56—C55	178.8 (3)

### 10-Phenyl-9-[2-(trifluoromethyl)phenyl]-3,4,6,7,9,10-hexahydroacridine-1,8(2H,5H)-dione Hydrogen-bond geometry (Å, º)

*Cg*, *Cg*5 and *Cg*13 are the centroids of the C2–C7, C21–C26 and C51–C56 rings, respectively.

**Table:**

*D*—H···*A*	*D*—H	H···*A*	*D*···*A*	*D*—H···*A*
C8—H8···F2	0.98	2.12	2.892 (4)	134
C38—H38···F4	0.98	2.15	2.908 (4)	133
C11—H11*B*···*Cg*2^i^	0.97	2.87	3.577 (5)	130
C13—H13*B*···*Cg*5	0.97	3.00	3.643 (4)	125
C46—H46*B*···*Cg*13	0.97	2.98	3.615 (4)	124
C1—F3···*Cg*13^ii^	1.33 (1)	3.93 (1)	4.949 (5)	134 (1)

Symmetry codes: (i) −*x*, −*y*+1, −*z*+2; (ii) −*x*, −*y*+2, −*z*+2.
